# Correlation between TCM Constitutional Types and Lung Carcinoma in Various Geographical Areas: A Systematic Review and Meta-Analysis

**DOI:** 10.1155/2022/5660231

**Published:** 2022-08-17

**Authors:** Xinyu Yu, Lei Yan, Qin Lan, Lilin Wan, Jun Xiong, Leichang Zhang, Huyun Nie, Zhaohui Ding

**Affiliations:** ^1^Center for Treatment of Diseases, Nanchang Hongdu Hospital of Traditional Chinese Medicine, Nanchang, Jiangxi, China; ^2^Ophthalmology Department, Jiangxi Integrated Hospital of Traditional Chinese and Western Medicine, Nanchang, Jiangxi, China; ^3^Pulmonary Disease Department, Affiliated Hospital of Jiangxi University of Traditional Chinese Medicine, Nanchang, Jiangxi, China; ^4^Department of Acupuncture and Moxibustion, Affiliated Hospital of Jiangxi University of Traditional Chinese Medicine, Nanchang, Jiangxi, China; ^5^Department of Proctology, Affiliated Hospital of Jiangxi University of Traditional Chinese Medicine, Nanchang, Jiangxi, China; ^6^Evidence-Based Medicine Research Centre, Jiangxi University of Traditional Chinese Medicine, Nanchang, Jiangxi, China

## Abstract

**Background:**

Lung carcinoma is a serious disorder that negatively influences the quality of life of sufferers. Despite the growing number of investigations into the management and prognosis of lung carcinoma, few research studies have been conducted to demonstrate the association between TCM constitution and lung carcinoma.

**Methods:**

We searched PubMed, EMBASE, Science Net, Cochrane Library, China National Knowledge Infrastructure, VIP database, Wanfang database, and China Biomedical Literature Database for Chinese and English versions until January 31, 2021. We also manually searched for Chinese lung cancer, Chinese physical medicine, Chinese medical trial registries, and unpublished surveys or references. The literature was screened against inclusive and exclusive criteria, and two investigators' results were independently summarized. The primary outcome was a ratio of body type. Single-group rates were meta-analyzed using Stata 14.0 statistical software, bias was estimated by funnel plotting, and sources of heterogeneity were evaluated by subgroup and sensitivity examinations.

**Results:**

18 randomized controlled trials were totally included to compare the single-group ratio and 95% confidence interval of nine constitution types of lung cancer, namely, mild constitution (ES = 0.12, 95% CI (0.08, 0.15), *P* < 0.0001), Qi deficiency constitution (ES = 0.20, 95% CI (0.15, 0.26), *P* < 0.0001), Qi depression constitution (ES = 0.09, 95% CI (0.07, 0.12), *P* < 0.0001), damp-heat constitution (ES = 0.05, 95% CI (0.03, −0.06), *P* < 0.0001), phlegm dampness constitution (ES = 0.05, 95% CI (0.03, −0.06), *P* < 0.0001), special constitution (ES = 0.01, 95% CI (0.01, 0.02), *P*=0.993), blood stasis constitution (ES = 0.05, 95% CI (0.04, 0.07), *P* < 0.0001), Yang deficiency constitution (ES = 0.16, 95% CI (0.12, 0.19), *P* < 0.0001), and Yin deficiency constitution (MD = 0.15, 95% CI (0.11, 0.18), *P* < 0.0001).

**Conclusion:**

This study showed that Qi deficiency, Yang deficiency, and Yin vacuity were the predominant types of physical conditions of lung cancer cases.

## 1. Introduction

The incidence rate of malignancies is the highest in the world. Lung cancer has one of the highest incidence rates of malignancy, whose death rate is higher than that of other carcinomas [1]. According to the latest national report on chronic diseases, approximately 700 thousand people in China have lung cancer each year, and about 600 thousand patients die [2]. In China, the incidence of newly diagnosed lung carcinoma patients and mortality from pulmonary carcinoma is 37% and 39.3%, respectively, ranking the highest in the world [3]. The disease burden is more serious; as a chronic disease, lung cancer creates a tremendous load on patients, their families, and communities [4]. Therefore, effective prevention and treatment of lung cancer have important social significance.

The etiology of lung cancer remains unclear and the clinical manifestations are complex. The clinical symptoms of the patients are mild and without clear discomfort. Therefore, most patients are diagnosed in the middle or late stages, thereby missing the optimal period for therapy [5].

Therefore, early identification and screening of lung cancer are of clinical importance. Traditional Chinese medicine has specialized in “preventing and treating sickness” since ancient times. Physique identification as an evaluation system was released in the “Classification of Physique in Chinese Medicine” released by the Chinese Academy of Traditional Chinese Medicine in 2009 [6].

To systematically analyze the classification of lung cancer constitution, we collected the relevant clinical studies published recently. We aimed to determine the classification rules of lung cancer constitution, provide effective proof of evidence-based medicine for clinical prevention and treatment, achieve early detection and early treatment, and establish a conceptual underpinning for medical management and cure.

## 2. Materials and Methods

### 2.1. Systematic Review and Registration

The registry code of this review was INPLASY202110030 (https://inplasy.com/inplasy-2021-1-0030/). Preferred reporting items for systematic review and meta-analysis statement (PRISMA) were utilized.

### 2.2. Literature Search

From December 1, 2020 to January 31, 2021, we searched PubMed, EMBASE, Science Net, Cochrane Library, China National Knowledge Infrastructure, VIP database, Wanfang database, and China Biomedical Literature database in Chinese and English. In addition, we manually searched for Chinese lung cancer, TCM constitution, China Medical Trial Registry, and nonpublished investigations or referencing. A research methodology was built using key phrases and clinical topic heading terms was (“Lung Neoplasms” [MeSH] OR “lung tumor” OR “lung cancer”) AND “constitution” [tiab] AND (“Medicine Chinese Traditional” [MeSH] OR “Chinese medicine” [tiab]). Retrieval policies for additional libraries were adapted to match their searching guidelines.

### 2.3. Inclusive Criteria

The research was deemed to be eligible when the below criterion was achieved:

1. Researched types. randomized controlled trials (RCTs) and cross-sectional studies were included in this review.
2. Researched object. lung cancer patients and the diagnosis should be based on the results of pathological examinations.
3. According to the standards of “2021 ESMO, European Society for Medical Oncology small-cell lung carcinoma: Practical medical directions for the identification, management, and subsequent monitoring of ESMO.” In 2009, the Chinese society of traditional Chinese medicine formulated the standard of TCM constitution classification and judgment. In 2021, the National Cancer Center and the China Lung Cancer Screening and Early Diagnosis and Early Treatment guideline development advisory group formulated lung cancer screenings, early diagnosis, and treatment guidelines in China.
4. The experimental group was an abnormal constitution (including mild constitution, Qi deficiency constitution, Qi depression constitution, damp-heat constitution, phlegm dampness constitution, special constitution, blood stasis constitution, Yang deficiency constitution, and Yin deficiency constitution) and the control group was a normal and peaceful constitution.

### 2.4. Exclusive Criteria

Studies with the below characteristics were eliminated:

1. Researched type, review, meta-analysis, comments, letters from readers, case reports, case series analysis, animal experiments, toxicological or pathological research, and nonmedicinal research.
2. Studies that failed to provide complete information.

### 2.5. Outcome

The outcomes of this systematic evaluation included the incidence of lung cancer in patients with different constitutions.

### 2.6. Data Extraction and Quality Assessment

After examining the duplicates of related studies, two researchers (Lan and Wang) screened them in two steps by inclusive and exclusive standards. The first step was to conduct a preliminary screening depending on the heading, summary, and keywords to determine whether they were included; the second step was to view the entire articles to confirm the ultimate eligible research for inclusion and exclusion. If there was a difference, it was negotiated or judged by a senior researcher (Yu). The standardized tables were used to extract relevant information from the literature: (1) research title, author(s), and year of publication; (2) research field, sample size, and researched type; and (3) case source and basic patient information.

We used the criteria suggested by Agency for Healthcare Research and Quality (AHRQ) to evaluate included studies. The AHRQ used 11 items to evaluate data sources, observation time, and quality control. If the answer to an item was “no” or “do not know,” no point was scored; if the answer was “yes,” 1 point was scored. The research was rated at three quality levels, namely, poor (0 to 3 marks), medium (4 to 7 marks), and high (8 to 11 marks) [7].

The research evaluation process was undertaken by two independent contributors (Lan and Wang). The third author finally decided on the research with inconsistent conclusions (Yu).

### 2.7. Data Synthesis

Single-group rate analysis in the meta-package of Stata 14.0 was adopted for data analysis. The rate and 95% confidence interval (CI) of different TCM physique types in the lung cancer population were calculated, and the main result was displayed in the forest map. The *I*^2^ value was used to determine the heterogeneity between studies. If *I*^2^ > 50%, the heterogeneity was considered statistically significant. Subgroups and sensitivities should be undertaken to examine for possible origins of heterogeneity, such as regional variations. The sensitivity analysis was carried out by excluding relevant studies for each body type separately. Then, it reanalyzed and observed the heterogeneity. Finally, a funnel chart was used for bias assessment [7–9].

## 3. Results

### 3.1. Literature Search Results

**Figure 1:**
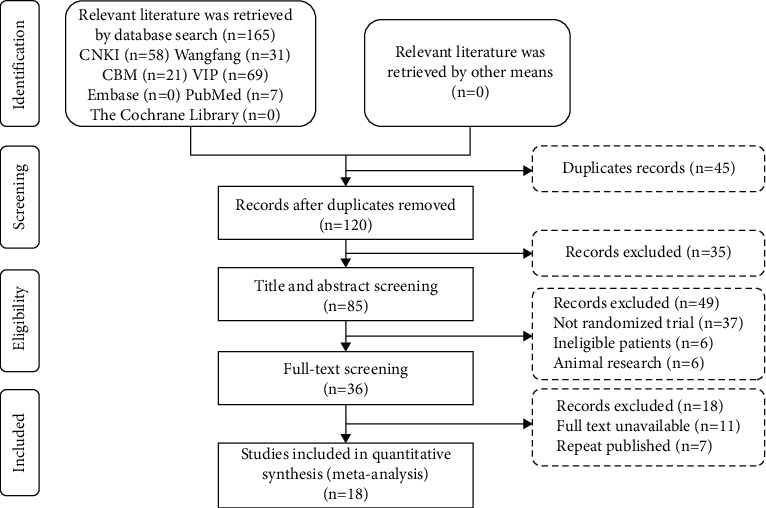
Article screening flow chart and options for research.

According to the PICOS principle, we identified a total of 165 studies. After deleting duplicate studies, we identified and read 120 studies. After screening the studies twice, we excluded 102 studies, such as pathology, animal, statistical analysis, and nonlung cancer studies. Finally, a total of 18 RCTs [10–26] were selected (Figure 1 and Table 1).

### 3.2. Measurement Indicators

The included studies were all cross-sectional surveys published from 2009 to 2020. The research areas included 5 regions in Eastern China, Northern China, Central China, Southern China, and Northwest China. The cases came from outpatients and inpatients in hospitals. In all included literature, pathological examinations were used as the diagnostic criteria. In addition, the pathological types of the included patients were reported.

## 4. Meta-Analysis Outcomes

A large heterogeneity existed across the results of the studies, and thus random-effect models were used for data analysis. The quality ratio of calmness, Qi deficiency, Yang deficiency, and Yin deficiency in the lung cancer patient population was larger. A subgroup analysis was performed for each constitution type according to geographical division.

### 4.1. Mild Constitution

**Figure 2:**
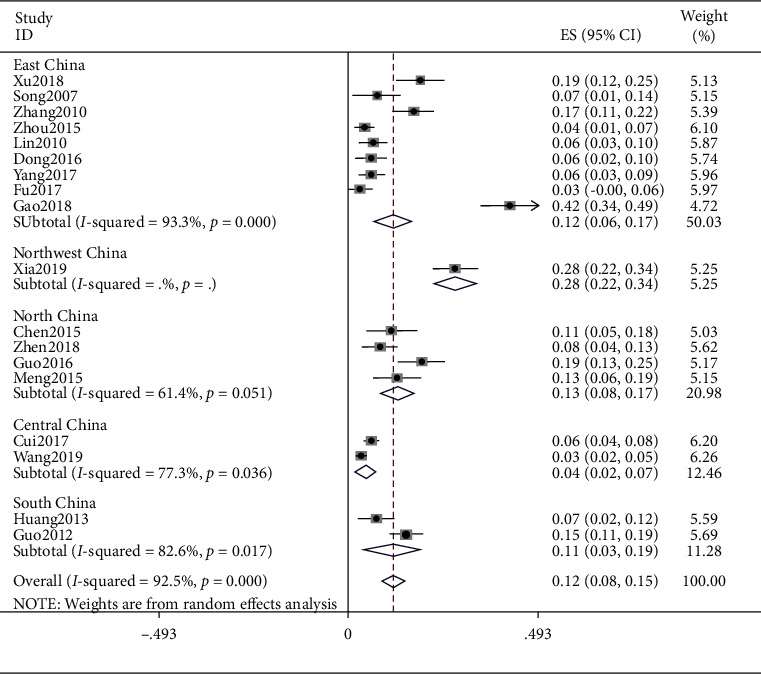
Meta-analysis of data on peace quality.

The meta result of peace quality was (ES = 0.12, 95% CI (0.08, 0.15), *p* < 0.0001) and that of East China was (ES = 0.12, 95% CI (0.06, 0.17), *p* < 0.0001). The meta result of Northeast China was (ES = 0.28, 95% CI (0.22, 0.34), *p* < 0.0001), North China was (ES = 0.13, 95% CI (0.08, 0.17), *p*=0.051), Central China was (ES = 0.04, 95% CI (0.02, 0.07), *p*=0.036), and South China was (ES = 0.11, 95% CI (0.03, 0.19), *p*=0.017). Detailed observations are shown in Figure 2.

### 4.2. Qi Deficiency

**Figure 3:**
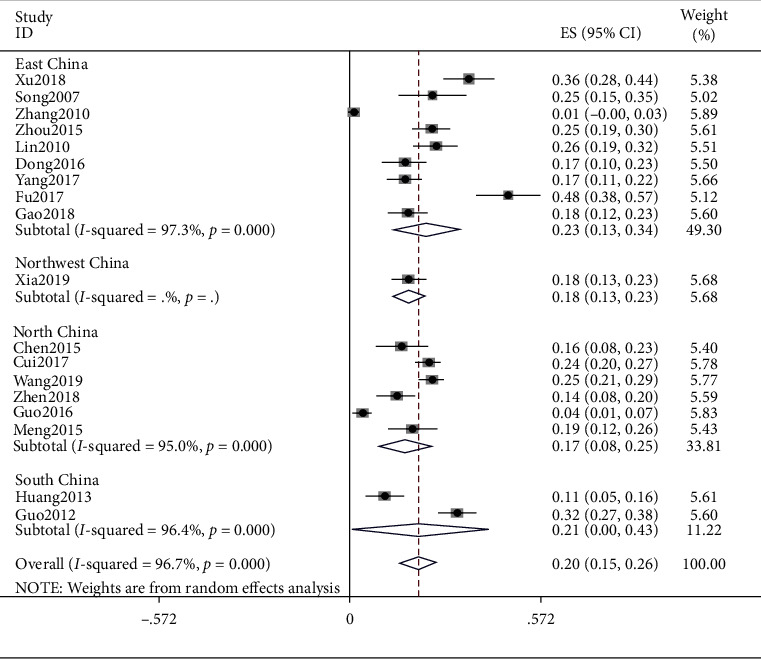
Meta-analysis of Qi deficiency.

The results of the study showed that the meta result of Qi deficiency was (ES = 0.20, 95% CI (0.15, 0.26), *p* < 0.0001) and that of East China was (ES = 0.23, 95% CI (0.13, 0.34), *p* < 0.0001), Northeast China was (ES = 0.18, 95% CI (0.13, 0.23), *p* < 0.0001), North China was (ES = 0.17, 95% CI (0.08, 0.25), *p* < 0.0001), and South China was (ES = 0.21, 95% CI (0.15, 0.26), *p* < 0.0001). Detailed data are shown in Figure 3.

### 4.3. Yang Deficiency

**Figure 4:**
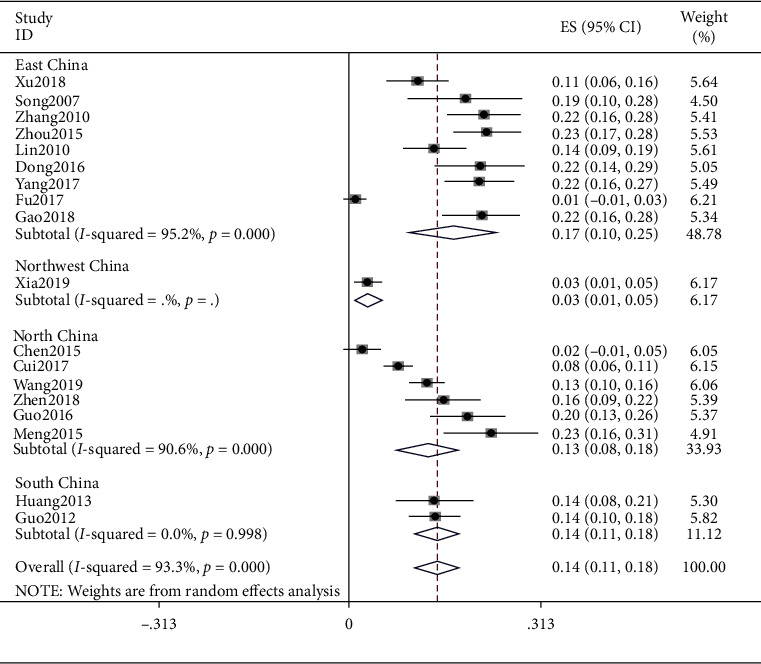
Meta-analysis of Yang deficiency.

The meta result of Yang deficiency was (ES = 0.14, 95% CI (0.11, 0.18), *p* < 0.0001) and that of East China was (ES = 0.17, 95% CI (0.10, 0.25), *p* < 0.0001), Northeast China was (ES = 0.03, 95% CI (0.01, 0.05), *p* < 0.0001), North China was (ES = 0.13, 95% CI (0.08, 0.18), *p* < 0.0001), and South China was (ES = 0.14, 95% CI (0.11, 0.18), *p* < 0.0001). Detailed observation is shown in Figure 4.

### 4.4. Yin Deficiency

**Figure 5:**
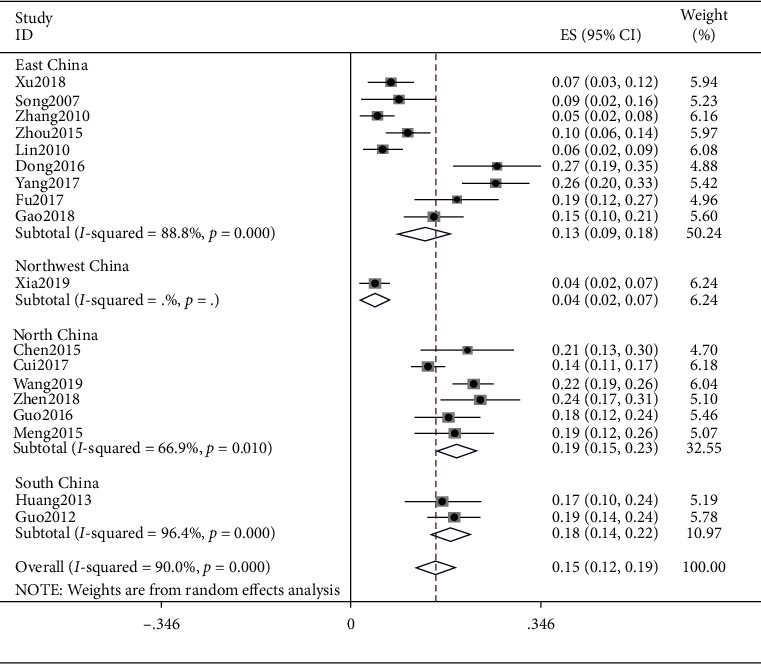
Meta-analysis of Yin deficiency.

The result of Yin deficiency was (ES = 0.15, 95% CI (0.12, 0.19), *p* < 0.0001) and that of East China was (ES = 0.13, 95% CI (0.09, 0.18), *p* < 0.0001), Northeast China was (ES = 0.04, 95% CI (0.02, 0.07), *p* < 0.0001), North China was (ES = 0.19, 95% CI (0.15, 0.23), *p*=0.0010), and South China was (ES = 0.15, 95% CI (0.14, 0.22), *p*=0.638). Detailed results are shown in Figure 5.

### 4.5. Qi-Stagnation Constitution

**Figure 6:**
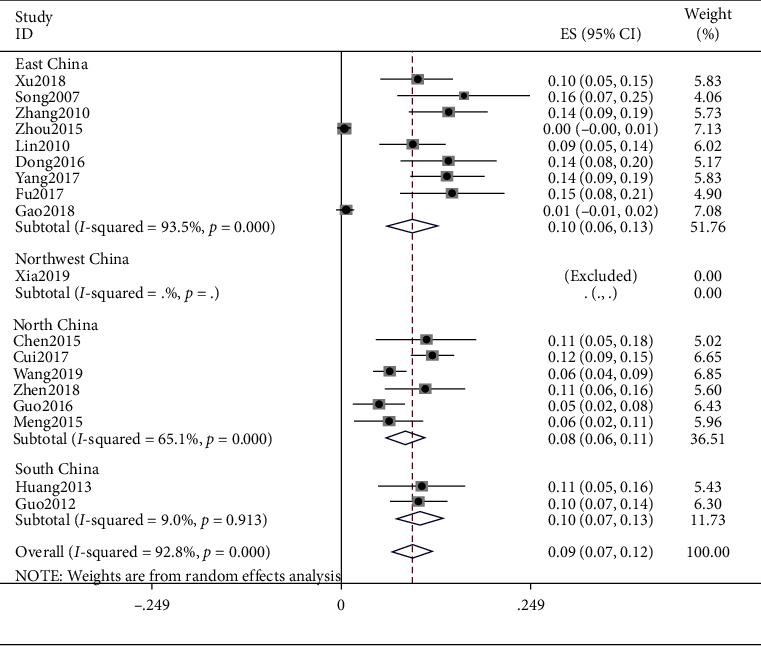
Meta-analysis of data on Qi-stagnation constitution.

The meta result of Qi-stagnation constitution was (ES = 0.09, 95% CI (0.07, 0.12), *p* < 0.0001) and that of East China was (ES = 0.10, 95% CI (0.06, 0.13), *p* < 0.0001), North China was (ES = 0.08, 95% CI (0.06, 0.11), *p*=0.008), and South China was (ES = 0.10, 95% CI (0.07, 0.13), *p*=0.913). Detailed observations are shown in Figure 6.

### 4.6. Damp-Heat Constitution

**Figure 7:**
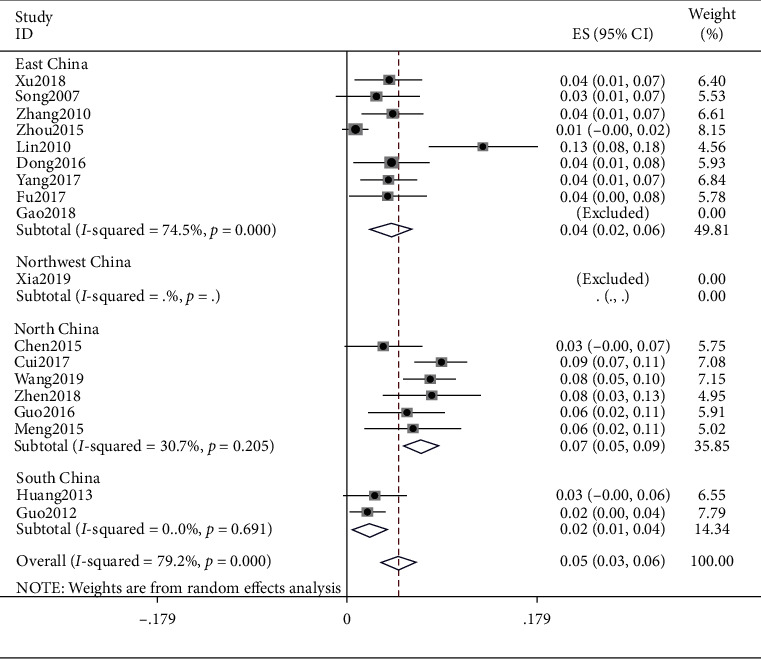
Meta-analysis of damp-heat constitution.

The meta result of damp-heat constitution was (ES = 0.05, 95% CI (0.03, 0.06), *p* < 0.0001) and that of East China was (ES = 0.04, 95% CI (0.02, 0.06), *p* < 0.0001), North China was (ES = 0.07, 95% CI (0.05, 0.09), *p*=0.205), and South China was (ES = 0.02, 95% CI (0.01, 0.04), *p*=0.691). Detailed observations are shown in Figure 7.

### 4.7. Phlegm Dampness

**Figure 8:**
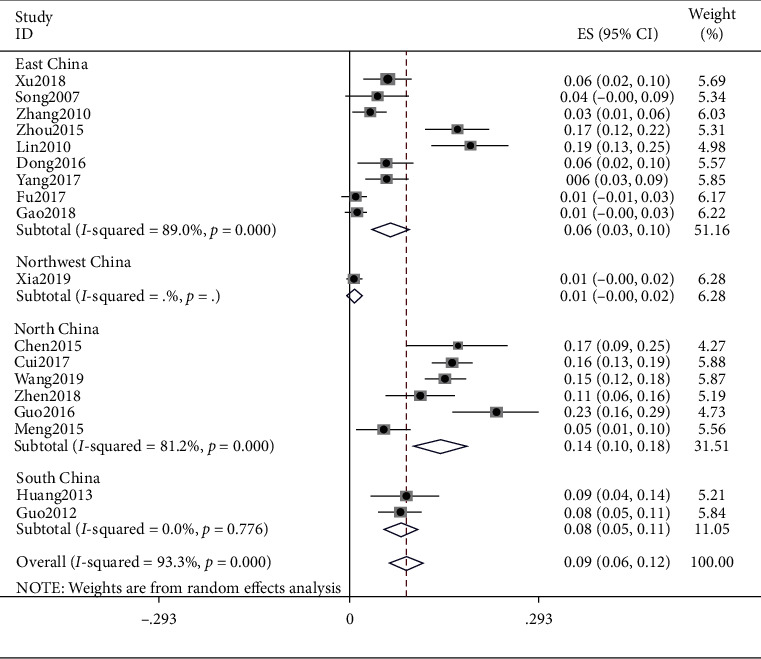
Meta-analysis of phlegm dampness.

The meta result of phlegm dampness was (ES = 0.05, 95% CI (0.03, 0.10), *p* < 0.0001) and that of East China was (ES = 0.04, 95% CI (0.02, 0.06), *p* < 0.0001), Northwest China was (ES = 0.01, 95% CI (−0.00, 0.02)), Northern China was (ES = 0.14, 95% CI (0.10, 0.18), *p* < 0.0001), and South China was (ES = 0.08, 95% CI (0.06, 0.11), *p*=0.776). Detailed observations are shown in Figure 8.

### 4.8. Blood Stasis

**Figure 9:**
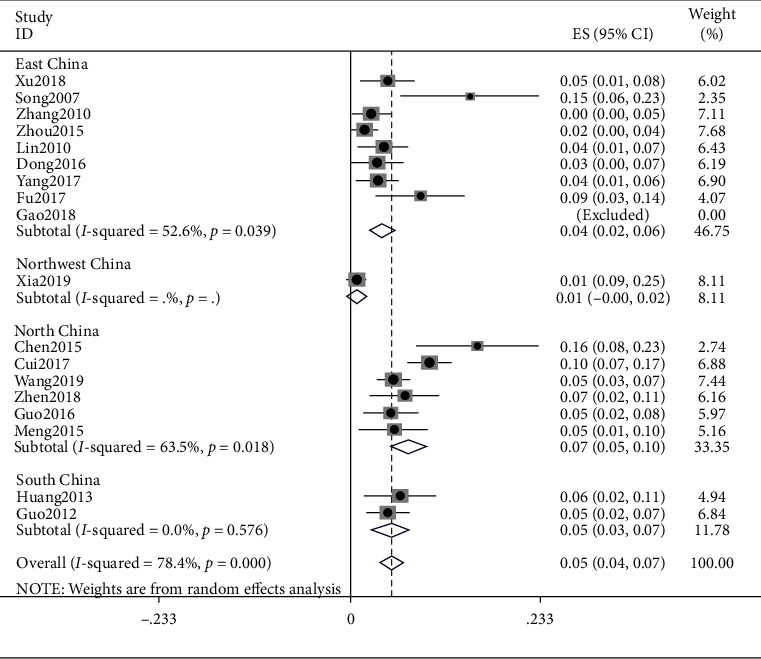
Meta-analysis of data on blood stasis.

The meta result of blood stasis was (ES = 0.05, 95% CI (0.04, 0.07), *p* < 0.0001) and that of East China was (ES = 0.04, 95% CI (0.02, 0.06), *p*=0.039), Northwest China was (ES = 0.01, 95% CI (−0.00, 0.02)), Northern China was (ES = 0.07, 95% CI (0.05, 0.10), *p*=0.018), and South China was (ES = 0.05, 95% CI (0.03, 0.07), *p*=0.576). Detailed observations are shown in Figure 9.

### 4.9. Special Constitution

**Figure 10:**
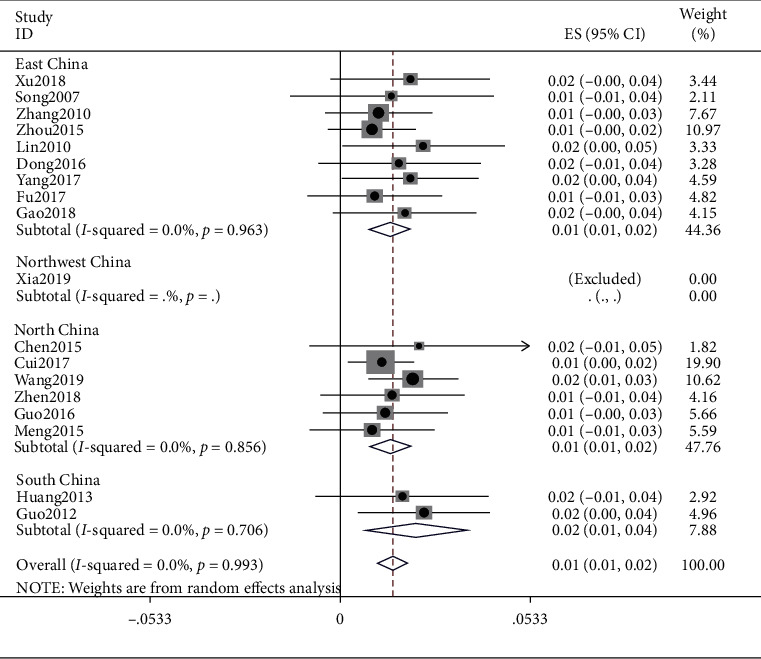
Meta-analysis of data on special constitution.

The meta result of special constitution was (ES = 0.01, 95% CI (0.01, 0.04), *p*=0.706) and that of East China was (ES = 0.01, 95% CI (0.01, 0.02), *p*=0.963), Northwest China was (ES = 0.01, 95% CI (−0.00, 0.02)), North China was (ES = 0.01, 95% CI (0.01, 0.02), *p*=0.856), and South China was (ES = 0.02, 95% CI (0.01, 0.04), *p*=0.706). Detailed observations are shown in Figure 10.

## 5. Subgroup Analysis

According to the subgroup analysis based on the region, there were 9 items in East China, 1 item in Northeast China, 6 items in North China, and 2 items in South China. The constitution type of lung cancer patients in East China from high to low was Qi deficiency constitution (ES = 0.23, 95% CI (0.13,0.340), *p* < 0.0001), Yang deficiency constitution (ES = 0.17, 95% CI (0.10, 0.25), *p* < 0.0001), Yin deficiency constitution (ES = 0.13, 95% CI (0.09, 0.18), *p* < 0.0001), mild constitution (ES = 0.12, 95% CI (0.06,0.17),*p* < 0.0001), Qi-stagnation constitution (ES = 0.10, 95% CI (0.06, 0.13), *p* < 0.0001), phlegm dampness constitution (ES = 0.05, 95% CI (0.03, 0.10), *p* < 0.0001), damp-heat constitution (ES = 0.04, 95% CI (0.02, 0.06), *p* < 0.0001), blood stasis (ES = 0.04, 95% CI (0.02, 0.06), *p*=0.039), and special constitution (ES = 0.01, 95% CI (0.01, 0.02), *p*=0.963). The constitution type of lung cancer patients in North China from high to low was Yin deficiency constitution (ES = 0.19, 95% CI (0.15, 0.23), *p*=0.010), Qi deficiency constitution (ES = 0.17, 95% CI (0.08, 0.25), *p* < 0.0001), phlegm constitution (ES = 0.14, 95% CI (0.10, 0.18), *p* < 0.0001), mild constitution (ES = 0.13, 95% CI (0.08,0.17), *p*=0.051), Yang deficiency constitution (ES = 0.13, 95% CI(0.08, 0.18), *p* < 0.0001), Qi-stagnation constitution (ES = 0.08, 95% CI (0.06, 0.11), *p*=0.0008), damp-heat constitution (ES = 0.07, 95% CI (0.05, 0.09), *p* < 0.0001), blood stasis constitution (ES = 0.07, 95% CI (0.05, 0.10), *p*=0.018), and special constitution (ES = 0.01, 95% CI (0.01, 0.02), *p*=0.856). The constitution type of lung cancer patients in South China from high to low was Qi deficiency constitution (ES = 0.21, 95% CI (0.01, 0.43), *p* < 0.0001), Yin deficiency constitution (ES = 0.15, 95% CI (0.12, 0.19), *p*=0.638), Yang deficiency constitution (ES = 0.14, 95% CI (0.11, 0.18), *p*=0.998), mild constitution (ES = 0.11, 95% CI (0.03, 0.19), *p*=0.017), Qi depression constitution (ES = 0.10, 95% CI (0.07, 0.13), *p*=0.913), phlegm constitution (ES = 0.08, 95% CI (0.05, 0.11), *p*=0.776), blood stasis constitution (ES = 0.05, 95% CI (0.03, 0.07), *p*=0.576), damp-heat constitution (ES = 0.02, 95% CI (0.01, 0.04), *p*=0.691), and special constitution (ES = 0.02, 95% CI (0.01, 0.04), *p*=0.706).

## 6. Publication Bias

**Figure 11:**
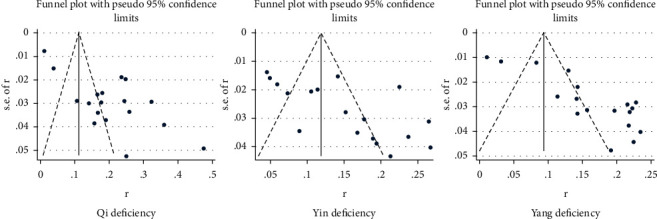
Funnel analysis chart.

A funnel chart was used to analyze the publication bias of the three constitution types of Qi deficiency, Yin deficiency, and Yang deficiency. The results suggested that the distribution of points in the funnel chart was poorly symmetric. The distribution of research points was asymmetrical, and the existence of publication bias was clear. Detailed observation is shown in Figure 11.

## 7. Discussion

The incidence rate of lung carcinoma is high worldwide, which seriously threatens the life safety of the population (27]. The previous research on various diseases and the causes of related diseases in traditional Chinese medicine determined the incidence of patients by the damage of disease pathogens to the body and their own physical strength. Patients with different TCM constitutions have different resistance to different types of diseases and different degrees of pathogenic stimulation [28]. The study found that the imbalance of Qi, Yang, and Yin in the human body and the lack of healthy qi were also causes of the disease. In the research on TCM constitution for the human body, TCM constitution can be divided into nine different types, and each TCM constitution group has its own characteristics [29]. This study studied the correlation between lung cancer patients and traditional Chinese medicine constitution frequently applied in TCM. The illness' internal cause is closely related to the constitution, which determines whether the human body feels evil or not to a certain extent. The physical condition determines the strength of the healthy qi of the human body, and different physical situations contribute decisively to the susceptibility and predisposition to tumors.

### 7.1. Analysis of TCM Constitution Types of Patients with Lung Cancer

It was found that Qi deficiency, Yin deficiency, and Yang deficiency were the three main constitution types for lung cancer patients, among which Qi deficiency was the first and foremost constitution. Currently, the prevalence of lung cancer in China is increasing year-by-year [30]. The large-scale cross-sectional study of traditional Chinese medicine constitution by Professor Wang Qi found that the proportion of mild constitution is greater. The results released that people with Qi deficiency had a higher risk of illness. The reciprocal damage of Yin and Yang tended to spread for a long time in these patients. In addition, the Qi-stagnation constitution is only the constitution of Yin deficiency and Yang deficiency, indicating that the incidence of lung cancer is related to emotional factors [31]. TCM maintains that grief and sorrow hurt the lungs. In addition, western medicine regards persistent negative emotions as a risk factor for cancer, thereby severely reducing the well-being and treatment effects of lung cancerous cases. Therefore, we must constantly understand the physical characteristics of lung cancer patients. On top of this, it has improved the patient's physical fitness and regulates the patient's lung function throughout the treatment and prevention of lung cancer [32].

In this study, it was also found that there were differences in the constitution types of lung cancer in different regions of China. Qi deficiency has been dominant in East China but is dominated by Yin deficiency and Yang deficiency. In North China, Yin deficiency has been the main type, followed by Qi deficiency and phlegm dampness [33]. Qi deficiency has also been dominant in South China but is dominated by Yang deficiency and Yin deficiency. TCM physique believes that physique is a relatively stable individual characteristic but also has variability. The confirmation of physique adjustability research showed that we could start with improving physique and restoring the individual pathological state of lung cancer patients [34]. The study found that the clinical constitution types of lung cancer patients were mainly peace, which Qi deficiency, yin deficiency and yang deficiency, and the frequency of qi deficiency was higher. Serum-related cytokines increased in varying degrees, suggesting a correlation between physique and the internal differences of diseases. It was feasible to adjust physique through drugs. In addition, the theory of “distinguishing body and using prescription” put forward by Wang Qi et al. was based on people's physical types (including Yin deficiency, Yang deficiency, Qi deficiency, phlegm and dampness, and other different constitutions) and stated (including distinguishing the strength of physique, fat and thin, age, north and south residence, and nursing advantages and disadvantages), so as to increase the fineness and individual pertinence of clinical prescriptions and then improve the curative effect of medication [35]. Therefore, in the prevention and treatment of clinical lung cancer, the biased constitution of patients should be fully considered, and the treatment time and dose ratio of attacking and righting drugs should be balanced.

### 7.2. Problems in Current Research and Suggestions for Improvement

As a cross-sectional study, this research did not follow the international statement of reporting of observational studies in epidemiology (STROBE). The overall report of this study lacked standard formatting, which was mainly reflected in the incomplete collection of information reports for many studies, such as the time of the study, gender, and treatment [36]. To a certain extent, the results were biased and it was difficult to conduct follow-up research. A rigorous feasibility plan should be formulated in future research by following relevant STROBE standards. At the same time, the research report should clearly inform the patient's basic information, treatment intervention measures, and pathological classification to facilitate further research.

### 7.3. Limitations of This Study

Despite our research on the relationship between age, sex, pathological type, and constitution type of the included studies, the incomplete information of the included subjects increased the heterogeneity and the risk of bias. Second, the experimental sites of all original studies included in this study are in China. There was a lack of research in the northwest and southwest regions, which required further studies.

## 8. Conclusion

Our research found that Qi deficiency, Yang deficiency, and Yin deficiency are the main constitution types of lung cancer patients, among which Qi deficiency has the closest relationship with lung cancer. However, due to nonstandard research and insufficient data reporting, more rigorous empirical medical research is needed to draw better conclusions.

## Figures and Tables

**Table 1:** The features of the enrolled researches.

Study	Area	Design	Sample	Participant	Average	Gender ratio	Types of lung neoplasms
Xu [14]	A	Cross-sectional study	150	A	54.54 ± 11.35	2 : 1	A
Song [22]	A	Cross-sectional study	68	B	45.46 ± 12.35	2 : 3	NA
Xia [12]	B	Cross-sectional study	150	A	57.93 ± 15.48	2 : 1	NA
Zhang [26]	A	Cross-sectional study	184	A + B	61.46 ± 12.00	3 : 1	NA
Zhou 2015	A	Cross-sectional study	220	B	56.66 ± 9.52	3 : 2	B, C
Chen [20]	C	Cross-sectional study	89	A + B	67.98 ± 6.99	2 : 1	C, D
Lin [25]	A	Cross-sectional study	170	B	63.56 ± 7.13	3 : 1	C, D, E, F
Cui [16]	C	Cross-sectional study	512	B	64.23 ± 9.34	3 : 1	C, D, E, G
Wang [6]	C	Cross-sectional study	481	B	61 ± 10.3	1 : 1	C, D, G
Zhen [14]	C	Cross-sectional study	135	B	65.35 ± 4.32	2 : 1	B, F
Dong [19]	A	Cross-sectional study	120	A + B	64.45 ± 12.33	3 : 1	B
Yang [15]	A	Cross-sectional study	200	B	61.31 ± 3.24	2 : 1	F, C, D, G, E
Fu [17]	A	Cross-sectional study	103	A + B	59.39 ± .93	1 : 2	C, F, D
Guo [18]	C	Cross-sectional study	158	A + B	56.83 ± 6.87	1 : 2	NA
Huang [23]	D	Cross-sectional study	114	A	64.56 ± 7.46	3 : 2	C, D, E, F
Meng [21]	C	Cross-sectional study	111	A + B	64.59 ± 6.42	2 : 1	C, D
Guo [27]	D	Cross-sectional study	254	B	65.36 ± 8.34	1 : 2	E, F, C, D
Gao [13]	A	Cross-sectional study	165	B	62.1 ± 7.8	3 : 1	C, D
Xu [14]	A	Cross-sectional study	150	A	54.54 ± 11.35	2 : 1	NA

Notes: area : A : Eastern China; B : Northwest China; C : Northern China; and D : South China. A: outpatient and B: hospitalized patient. Types of lung neoplasms: A : pulmonary nodules; B : NSCLC; C: glandular cancer; D : SqCa; E : LCNEC; F: small cell carcinoma; and G : AdCa.

## Data Availability

The datasets used and/or analyzed during the current study are available from the corresponding author on reasonable request.

## Abbreviations

TCM: Traditional Chinese medicine
NSCLC: Nonsmall-cell lung carcinoma
SqCa: Squamous cell carcinoma
AdCa: Adenosquamous carcinoma
LCNEC: Large-cell carcinoma
PRISMA: Preferred reporting items for systematic review and meta-analysis protocols

## Supplementary Materials

Supplementary Materials Additional files 1: PRISMA 2009 checklist.
